# Venetoclax combined with hypomethylating agents and the CAG regimen in relapsed/refractory AML: a single-center clinical trial

**DOI:** 10.3389/fimmu.2023.1269163

**Published:** 2023-11-20

**Authors:** Yifan Liu, Yanfen Li, Ran Zhang, Zhangyu Yu, Yu Jing

**Affiliations:** Medical School of Chinese PLA, Department of Hematology in the Fifth Medical Center of PLA General Hospital, Beijing, China

**Keywords:** venetoclax, hypomethylating agents, CAG regimen, relapsed/refractory, acute myeloid leukemia

## Abstract

**Objective:**

This study aimed to evaluate the efficacy and safety of venetoclax in combination with hypomethylating agents and CAG (VEN-DCAG) regimens in patients with relapsed/refractory acute myeloid leukemia (R/R AML).

**Methods:**

The treatment response was analyzed by retrospective methods in R/R AML patients treated with the VEN-DCAG regimen at our institution. This included, but was not limited to, CR/CRi (complete remission/complete remission with incomplete hematologic recovery) rate, measurable residual disease (MRD) negative rate, and overall survival (OS).

**Results:**

20 patients with R/R AML were recruited, with a median age of 40 years (10-70), 11 of whom were male (55%), and a median follow-up of 10.4 months (0.7-21.8). The overall response rate (ORR) after receiving 1 course of VEN-DCAG was 90% (18/20), with 17 (85%) CR/CRi (10 MRD-CR), 1 (5%) PR, and 2 (10%) NR. Subsequently, 12 patients (7 MRD-CR, 4 MRD+CR, 1 NR) were treated with the VEN-DCAG regimen, and 3 MRD+CR patients turned negative, and 13 patients finally achieved MRD-CR. Among them, 7 patients were in the relapse group, all achieving CR/CRi (6 MRD-CR), and 13 patients in the refractory group, with 10 CR/CRi (7 MRD-CR). The ORR for patients in the relapse and refractory groups was 100% (7/7) and 84.6% (11/13), respectively. Further, all patients experienced adverse events (AEs) of varying degrees of severity, with hematologic AEs primarily consisting of myelosuppression, while non-hematologic AEs were more common in the form of fever, gastrointestinal distress, and infections. 11 patients were followed up with bridging allogeneic hematopoietic stem cell transplantation (allo-HSCT) therapy. At the last follow-up, 11 patients (7 MRD-CR, 4 MRD+CR) who received allo-HSCT, 1 (MRD+CR) died, and 9 patients (6 MRD-CR, 1 PR, 2 NR) who did not receive allo-HSCT, 5 (2 MRD-CR, 1 PR, 2 NR) died as well.

**Conclusion:**

The VEN-DCAG regimen may be an effective treatment option for R/R AML patients, with high ORR and MRD negative remission rates in both the relapsed and refractory groups. It is recommend that patients should be bridged to allo-HSCT as soon as possible after induction to CR by the VEN-DCAG regimen, which can lead to a significant long-term survival benefit.

**Clinical trial registration:**

https://www.chictr.org.cn/, identifier ChiCTR2300075985.

## 1 Introduction

Acute myeloid leukemia (AML) has a high relapse rate, and the prognosis after relapse is poor. The treatment measures are limited, and there is a lack of standardized treatment protocol (1, 2). In recent years, the use of targeted drugs and hypomethylating agents (HMAs) has expanded treatment options for patients with relapsed or refractory (R/R) AML, with some patients achieving complete remission, thus successfully bridging to allogeneic hematopoietic stem cell transplantation (allo-HSCT) and realizing the possibility of a cure for the disease. The Bcl-2 inhibitor venetoclax (VEN) is capable of targeting the anti-apoptotic protein Bcl-2, inducing apoptosis in tumor cells without relying on genetic mutations, and has shown great advantages in the treatment of hematologic malignancies (3–5).

Venetoclax in combination with HMAs has been approved as a first-line standard of care for elderly or unfit patients with newly diagnosed AML and has shown some efficacy in patients with R/R AML, although there is still need for more effective treatment options (6–8). The DCAG regimen, which combines hypomethylating agents with cytarabine, aclarubicin, and granulocyte colony-stimulating factor (G-CSF), has proven to be an effective treatment option for AML (9). Preclinical studies have demonstrated that venetoclax, when combined with decitabine and aclarubicin, has synergistic antitumor cell proliferative effects, promoting apoptosis and cell cycle arrest in AML cells (10, 11). Therefore, VEN-HMAs-CAG is likely to offer improved synergistic anti-AML cellular effects, however, there is a particular lack of clinical studies on this regimen. To further optimize the R/R AML treatment regimen and enhance the remission and survival rates of patients, this clinical trial analyzed the efficacy and safety of venetoclax combined with DCAG in the treatment of R/R AML patients.

## 2 Methods

### 2.1 Patient population

AML patients who had failed at least one cycle of intensive induction chemotherapy or developed relapse prior to receiving venetoclax combination therapy were eligible for this study. All enrolled patients were treated at our hospital between March 2021 and March 2023, voluntarily enrolled in this clinical trial, and provided informed consent on specified forms themselves or through their legal guardians. The patients were screened according to the following criteria: AML diagnosis was in accordance with the 5th edition of the World Health Organization (WHO) diagnostic criteria for 2016; patients had received at least 1 cycle of treatment with the VEN-DCAG regimen; an Eastern Cooperative Oncology Group (ECOG) score ≤3; ages between 10 and 70 years at the time of screening, regardless of gender; no severe allergies or psychiatric-related diseases; liver function: ALT or AST ≤ 2.5 times the upper limit of normal, bilirubin ≤ 2.0 times the upper limit of normal; renal function: creatinine within the upper limit of normal; no significant cardiac dysfunction (e.g., severe cardiac failure or cardiac arrhythmia); and no uncontrollable active infection. Exclusion criteria for patients included acute promyelocytic leukemia, pregnancy, breastfeeding, a history of autoimmune disease, chronic smoking or alcohol use that could interfere with trial evaluation, and investigator-determined unsuitability for the clinical trial (e.g., poor medical adherence, surgery within the last 6 weeks, etc.). All patients were diagnosed through bone marrow cytomorphology, leukemia immunophenotyping, cytogenetics, and molecular biology.

### 2.2 Study design and treatment regimen

This study aimed to explore the efficacy and safety of the combination of venetoclax, hypomethylating drugs, and CAG regimen in the treatment of R/R AML patients. It received approval from the Ethics Committee of the General Hospital of the People’s Liberation Army (S2022-740-01) and was registered as a clinical trial (ChiCTR2300075985).

Twenty patients with R/R AML were enrolled: 17 receiving the VEN + decitabine (DAC) + CAG regimen, and 3 receiving the VEN + azacitidine (AZA) + CAG regimen. The VEN administration consisted of 400 mg/d or 100 mg/d (combined with azole antifungals), d1-14, taken orally. DAC 20 mg/m^2^, d1-5, IV; AZA 50-75 mg/m^2^, d1-7, subcutaneously; CAG regimen: cytarabine 50-100 mg/m^2^/d, d1-5, IV; aclarubicin 20 mg/d (6 cases d1/3/5, 14 cases d1-5), IV; G-CSF 300 μg/d or polyethylene glycol long-acting colony-stimulating factor (PEG-CSF) 6 mg once a week; and G-CSF or PEG-CSF was discontinued when the white blood cell count (WBC) was ≥10×10^9^/L. Dose reduction in cytarabine or azacitidine were considered for patients with particular conditions: bone marrow hypoproliferation; advanced age (>60), or those unable to tolerate standard-dose chemotherapy, as well as individuals with heart, lung and kidney complications. Treatment was continued as long as the patient demonstrated clinical benefit, and symptomatic supportive treatment, such as blood products transfusion or anti-infection measures, was offered according to the actual situation.

### 2.3 Assessments

Patients were primarily assessed for 1-cycle efficacy and total efficacy, which included evaluating the complete remission/complete remission with incomplete hematologic recovery (CR/CRi) rate, partial remission (PR), no remission (NR), measurable residual disease (MRD) negative rate, overall survival (OS), event-free survival (EFS), and success rate of subsequent bridging HSCT. The safety assessment primarily included examining the occurrence of adverse events (AEs) taking place after VEN-DCAG treatment, with determinations made through clinical symptoms, laboratory tests, imaging examinations, and others. The adverse event severity grading standard was adopted from the National Cancer Institute (NCI) adverse event grading (CTC-AE) version 4.03 (12).

The main definitions are as follows: CR: Disappearance of signs and symptoms of leukemia, absence of leukemic cells in the leukocyte classification, <5% primitive cells in the bone marrow, absence of extramedullary leukemia, and neutrophil count ≥1.0×10^9^/L, PLT ≥100×10^9^/L. CRi: Disappearance of signs and symptoms of leukemia, absence of leukemic cells in the leukocyte classification, <5% primitive cells in the bone marrow, absence of extramedullary leukemia, and neutrophil count <1.0×10^9^/L or PLT <100×10^9^/L. PR: Bone marrow primitive cells decreased to 6%-20% or decreased over 50% from pretreatment. NR: Non-fulfillment of the above criteria. MRD negative status was defined as negative leukemia immunotyping by flow cytometry (FCM). Relapse was defined as the presence of >5% bone marrow primitive cells or the presence of extramedullary infiltrates after achieved CR (13). Refractory was defined as failure to achieve CR with at least one courses of previous-line induction chemotherapy. OS: The time from entry into VEN combination therapy until death from any cause or last follow-up. EFS: The time from entry into VEN combination therapy until disease progression, relapse, death from any cause, or last follow-up. The overall response rate (ORR) was calculated as CR + CRi + PR.

### 2.4 Follow-up and statistical methods

Patient statuses were acquired through telephone follow-ups and a review of outpatient or inpatient records. The follow-up cutoff date was April 1, 2023. The endpoint of the follow-up was either the follow-up cutoff or death from any cause. Data were analyzed using SPSS 26.0 software, and outcome data have been expressed as the median (range) or number (percentage). Survival curves were plotted using the Kaplan−Meier method, and survival was compared between groups using the log-rank test. A *P-value* < 0.05 was considered to indicate a statistically significant difference.

## 3 Results

### 3.1 Patient characteristics

**Figure 1:**
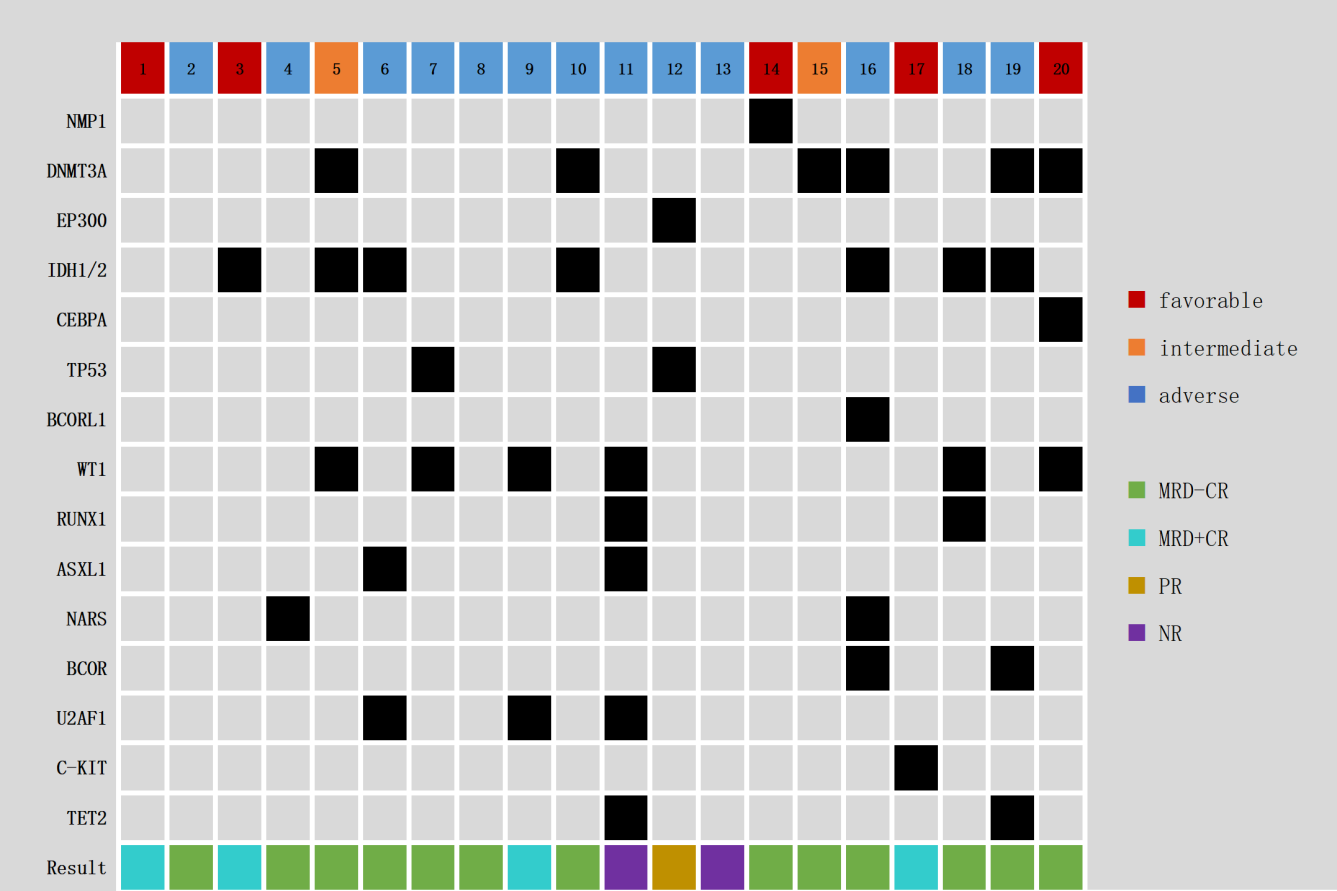
Genomic landscape in 20 R/R AML patients.

The median age of the 20 patients was 40 years (10-70), including 11 (55%) male and 9 (45%) female (**Table 1**). There were 17 cases of primary AML, 1 case was secondary to myelodysplastic syndrome (MDS); 1 case emerged 5 years after aplastic anemia unrelated donor transplantation secondary to AML, and 1 case pertained to treatment-related AML (anterior mediastinal germ cell tumor). The patients were grouped according to AML disease stage: 13 had refractory disease, 7 showed relapse, with 2 cases of relapse occurring after allo-HSCT. According to the ELN 2022 version of AML genetic risk stratification, 5 cases in the favorable prognosis group, 2 cases in the intermediate prognosis group, and 13 cases in the adverse prognosis group. Among these patients, 11 had received 1 course of previous treatment, 9 had received >1 course of treatment, 12 had received HMA treatment, 3 had received venetoclax, and 2 cases had received allo-HSCT. The median percentage of bone marrow (BM) primitive cells before treatment was 27.65 (4.0-76.8)%, the median hemoglobin (HGB) level was 78 g/L (45-135), the median white blood cell (WBC) count was 2.46 (0.59-64.51) × 10^9^/L, and the median platelet (PLT) count was 48 (6-873) × 10^9^/L. Chromosomal karyotyping showed 2 cases with a favorable prognosis karyotype, 13 cases with an intermediate prognosis karyotype, and 4 cases with an adverse prognosis karyotype. Reverse transcription RT−PCR molecular biology detected the AML1-ETO fusion gene in 3 cases each, the MLL-AF6 fusion gene and MLL-MLL fusion gene in 2 cases each, and the MLL-SEPTIN9 fusion gene and FUS-ERG fusion gene in 1 case each. Second-generation gene sequencing (NGS) detected 7 cases of IDH1/2 mutations, 6 cases each of DNMT3A and WT1 mutations, 3 cases of U2AF1 mutations, 2 cases each of NARS, ASXL1, TP53, BCOR, TET2, RUNX1, and 1 case each of BCORL1, NMP1, CEBPA, C-KIT and EP300 (nonsignificant mutations have been omitted) (**Figure 1**).

**Table 1:** Baseline characteristics of R/R AML patients treated with VEN-DCAG.

	VEN-DCAG (n=20)		
	Refractory group (n=13)	Relapse group (n=7)	All patients (n=20)
Median age, y (range)	41 (10-70)	39 (25-50)	40 (10-70)
Male sex, n (%)	8 (62)	3 (43)	11 (55)
Status before VEN-DCAG			
BM blast%, median (range)	28.5 (14.8-76.8)	26.8 (4.0-50.4)	27.7 (4.0-76.8)
WBC, 10^9^/L, median (range)	2.1 (0.6-64.5)	3.2 (1.4-3.5)	2.5 (0.6-64.5)
PLT, 10^9^/L, median (range)	28 (6-873)	113 (36-130)	48 (6-873)
AML type, n (%)			
M2	2 (15)	3 (43)	5 (25)
M4	3 (23)	1 (14)	4 (20)
M5 Other AML	3 (23) 5 (38)	2 (29) 1 (14)	5 (25) 6 (30)
Median prior therapies, n (range)	1 (1-3)	8 (1-9)	1 (1-9)
ELN 2022 risk group, n (%)			
Favorable	2 (15)	3 (43)	5 (25)
Intermediate	1 (8)	1 (14)	2 (10)
Adverse	10 (77)	3 (43)	13 (65)
Cytogenetic group, n (%)			
Normal karyotype	5 (38)	3 (43)	8 (40)
t (8;21)	2 (15)	1 (14)	3 (15)
Adverse-risk or complex	3 (23)	0 (0)	3 (15)
Other intermediate-risk	3 (23)	3 (43)	6 (30)

### 3.2 Efficacy

The ORR of 20 patients after 1 cycle of treatment was 90% (18/20), including 17 cases of CR/CRi (85%), 1 case of PR (5%), 2 cases of NR (10%), and 10 cases of MRD-CR (58.8%). Twelve patients (7 MRD-CR, 4 MRD+CR, 1 NR) received the follow-up VEN-DCAG regimen, with 10 patients undergoing 2 cycles, 1 patient completing 4 cycles, and 1 patient undergoing 5 cycles. Three of the 4 patients with 1-course MRD+CR were converted to MRD-CR after the 2nd course of VEN-DCAG, and 1 patient with NR had disease progression during the follow-up treatment. Finally, of the 20 patients, 17 achieved CR/CRi and 13 reached MRD-CR (76.5%) (**Table 2**).

**Table 2:** Final response outcomes for VEN-DCAG.

	Refractory group (n=13)	Relapse group (n=7)	All patients (n=20)
ORR, n (%)	11 (84.6)	7 (100.0)	18 (90.0)
CR/CRi	10 (76.9)	7 (100.0)	17 (85.0)
CR	7 (53.8)	5 (71.4)	12 (60.0)
CRi	3 (23.1)	2 (28.6)	5 (25.0)
MRD-CR	7 (70.0)	6 (85.7)	13 (76.5)
PR	1 (7.7)	0 (0.0)	1 (5.0)
NR	2 (15.4)	0 (0.0)	2 (10.0)
Risk group, CR rate, n^*^ (%)			
Favorable	2 (100.0)	3 (100.0)	5 (100.0)
Intermediate	1 (100.0)	1 (100.0)	2 (100.0)
Adverse	7 (70.0)	3 (100.0)	10 (76.9)
Bridging Allo-HSCT, n (%)	6 (46.2)	5 (71.4)	11 (55.0)
Median follow-up, median (range)	9.7 (0.7-21.8)	11.0 (2.6-20.9)	10.4 (0.7-21.8)

* Patients who did not achieve complete remission are not included in this sequence.

There were 7 patients in the relapse group, and all 7 patients achieved CR after 1 cycle, including 5 MRD-CR and 2 MRD+CR (1 MRD+CR was converted to MRD-CR with subsequent VEN-DCAG treatment). Among the two patients who had relapsed after allo-HSCT (both 5 years after transplantation), MRD-CR was reached after 1 cycle of VEN-DCAG. There were 13 patients in the refractory group, 10 patients (5 MRD-CR, 5 MRD+CR) achieved CR, 1 reached PR, and 2 witnessed NR after 1 cycle of treatment. Eight patients (4 MRD-CR, 3 MRD+CR, 1 NR) received follow-up treatment, of whom 2 MRD+CR patients converted to MRD-CR after the second cycle. 1 NR patient deteriorated during follow-up treatment. Eventually, the MRD negative remission rates in the relapse group and refractory group were 85.7% and 70.0%, respectively, with *P* > 0.05.

According to the ELN 2022 hazard stratified subgroup analysis, the MRD negative remission rates in the favorable prognosis, intermediate prognosis, and adverse prognosis groups were found to be 40.0%, 100.0%, and 90.0%, *P*=0.070, respectively, after treatment with VEN-DCAG. Further analysis of the differences in gene mutations’ effect on efficacy demonstrated no statistically significant variations between the different mutations due to the sample size limitations. Analyzing the effect of concomitant MLL fusion gene mutations on the efficacy, the results showed that the ORR with or without this fusion gene was 60.0% and 100%, *P*=0.053, and the CR rate was 60.0% and 93.3%, *P*=0.140, respectively. Following the chromosomal karyotype subgroup analysis results, the MRD negative remission rates after 1 course of treatment with this combination regimen in the favorable prognosis karyotype, intermediate prognosis karyotype, and complex karyotype groups were 100.0%, 25.0%, and 66.7%, respectively (*P*=0.011), and the final MRD negative remission rates were 100.0%, 50.0%, and 100.0%, respectively (*P*=0.086). Analysis of whether prior medication with VEN or HMAs affected efficacy highlighted that the receipt of HMAs was not significantly correlated with efficacy, whereas the ORR and CR rate were significantly higher in patients with no prior VEN treatment than in those who had received VEN treatment (ORR: 100.0% and 33.3%, *P*=0.016, CR rate: 94.1% and 33.3%, *P*=0.046). Further, analysis of whether age (≥50 years), disease type, and number of prior courses (≥2) affected efficacy showed no statistically significant difference in CR rate or MRD-CR rate.

Among the 3 patients without CR, there was 1 NR patient, a male, 70 years old, positive for MLL-AF6 fusion gene, 76% quantification of fusion gene, NR after 2 cycles of VEN-AZA treatment, NR after the 3rd course of VEN-DAC-HHT (homoharringtonine), and still NR after the 4th course adjusted to VEN-DCAG regimen, who finally died of disease progression. Another NR patient, a male, 37 years old, M4, with TET2, ASXL1 and U2AF1 mutations, VAF values of 50.95%, 35.78% and 42.47%, respectively, and positive MLL/MLL fusion gene, PR after the 1st course of VEN-DHAG, NR after the 2nd course of VEN-AZA-CAG, and still NR after the 3rd course of adjustment to VEN-DCAG, finally exhibited disease progression, multiple organ failure and death. A PR patient, a male, 20 years old, with an anterior mediastinal germ cell tumor with multiple metastases in the lung, was diagnosed with AML in the 2nd year of treatment by bone marrow (BM) examination due to thrombocytopenia. The chromosome examination revealed a complex karyotype (46-50, xy, +x [28], -4[28], del(5)(q31)[27], del(6)(q?23)[28], +9[27],?del(10)(p13)[5], +11[27], add(12)(q?24)[27],?add(15)(q22)[27], +21[20],+22[27][cp30]) and del(5q) positivity by FISH. The mutation sites detected by NGS were TP53 Exon8 c.817C>T p. R273C, TP53 Exon5 c.396G>T p. K132N, EP300 Exon6 c.1519A>G p. S507G, VAF values were 40.2%, 37.8%, 64.3%, respectively. There was NR after the 1st course of AZA-CAG and PR after the 2nd course of VEN-DCAG, but the anterior mediastinal tumor enlarged rapidly during treatment; both malignant tumors progressed, and the patient died during subsequent treatment.

### 3.3 Safety

Adverse events (AEs) were observed in all patients (**Table 3**). Grade ≥3 AEs were predominantly hematologic AEs, including leukopenia in 20 cases (100%), neutropenia in 20 cases (100%), thrombocytopenia in 19 cases (95%), and hemoglobin reduction in 19 cases (95%). The primary nonhematological AEs were 17 (85%) fevers in the granulocyte deficiency phase, 7 (35%) infections, including 4 cases of pneumonia, 2 skin and soft tissue infections, and 1 positive blood culture; 12 (60%) gastrointestinal discomfort reactions, including nausea, vomiting, diarrhea, abdominal pain, and constipation; 7 (35%) electrolyte disturbances, 4 (20%) skin rashes, 3 (15%) cases of skin/mucosal or nasal bleeding, and 2 (10%) cases each of abnormal liver function and weakness. The lowest median HGB value after treatment was 59.5 g/L (45.0-84.0), the lowest median WBC value was 0.22 × 10^9^/L (0.02-1.00), and the lowest median PLT value was 5 × 10^9^/L (1-97). Two NR patients had no signs of improvements after being given blood products and anti-infective treatment and died within 1 month after VEN-DCAG treatment while the remaining patients showed improvements after aggressive symptomatic treatment.

**Table 3:** Adverse events after treatment with VEN-DCAG.

All AEs, n (%)	All-grade (n=20)	≥3 Grade
Anemia	20 (100.0)	19 (100.0)
Thrombocytopenia	20 (100.0)	19 (95.0)
Neutropenia	20 (100.0)	20 (100.0)
Fever	17 (85.0)	9 (45.0)
Pneumonia	4 (20.0)	2 (10.0)
Skin/soft tissue infections	2 (10.0)	0 (0.0)
Bloodstream infections	1 (5.0)	0 (0.0)
Gastrointestinal symptoms	12 (60.0)	2 (10.0)
Electrolyte disorders	7 (35.0)	1 (5.0)
Mucosal/nasal bleeding	3 (15.0)	0 (0.0)
Rash	4 (20.0)	0 (0.0)
Liver function abnormalities	2 (10.0)	0 (0.0)
Fatigue	2 (10.0)	0 (0.0)

### 3.4 Follow-up and prognosis

The median cycle of VEN-DCAG treatment for all patients was 2 (1-5) cycles, with a median follow-up time of 10.4 (0.7-21.8) months and a median EFS of 9.2 (0.7-21.8) months. Eleven patients (7 MRD-CR, 4 MRD+CR) were subsequently bridged to allo-HSCT.

**Figure 2:**
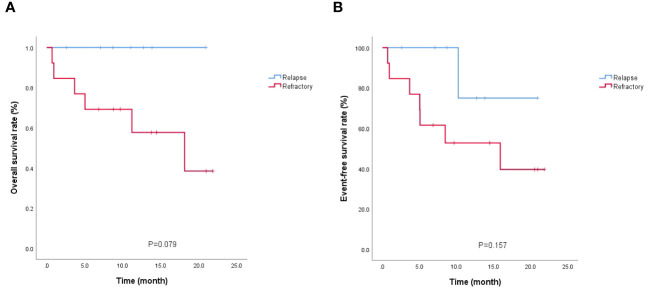
Survival analysis of relapsed/refractory patients. **(A)** OS; **(B)** EFS.

**Figure 3:**
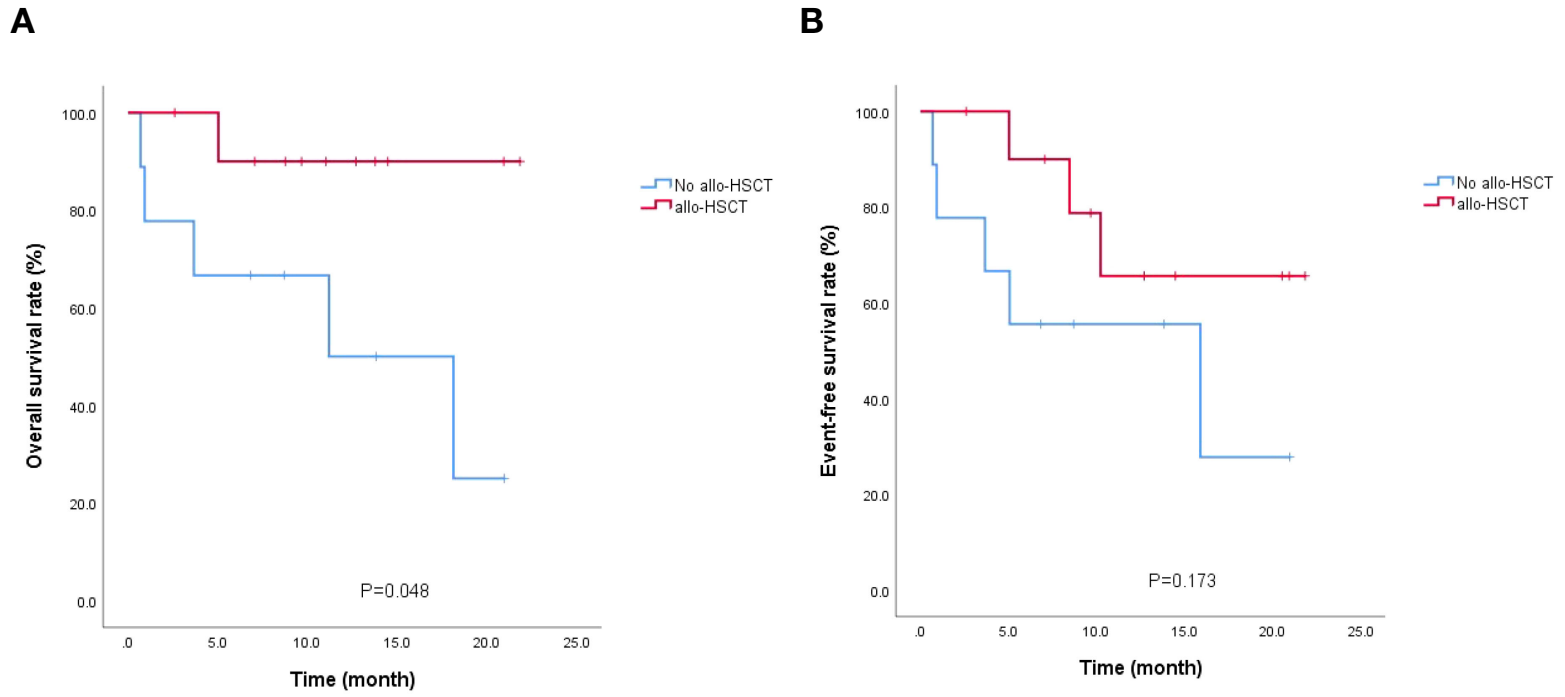
Survival analysis of bridging allo-HSCT/no allo-HSCT patients. **(A)** OS; **(B)** EFS.

By the end of follow-up, 14 of 20 patients survived (12 disease-free and 2 relapsed), with a median follow-up of 12.7 (2.6-21.8) months in 14 surviving patients; 6 died (1 after transplantation and 5 without transplantation), with a median OS of 4.35 (0.7-18.1) months in 6 deceased patients. 7 patients in the relapse group had no death and 6 of 13 patients in the refractory group died, *P*=0.051. Further, 10 of 11 patients who received allo-HSCT survived, and 4 of 9 patients who did not receive allo-HSCT survived (90.9% vs. 44.4%, *P*=0.05) (details in **Table S1**). Survival analysis showed no statistically significant difference (*P* > 0.05) in the comparison of OS and EFS between patients in the relapse and refractory groups (**Figure 2**) but a statistically significant difference (*P* = 0.048) in the comparison of OS between those who received allo-HSCT and those who did not receive allo-HSCT (**Figure 3**).

## 4 Discussion

Although multiple combination regimens have been effective in improving the remission rate of AML patients, nearly 50% of patients still fail to respond to treatment or relapse. R/R AML has a low remission rate and poor prognosis, with CR/CRi rates under conventional combination chemotherapy regimens of less than 40%, a 3-year overall survival of less than 10%, and a median survival of only 6 months (14–18). Currently, allo-HSCT is still the only treatment modality with curative potential. However, the majority of R/R AML patients lack opportunities of transplantation owing to the inability to obtain CR, or forcing transplantation with no CR severely weakens the transplantation outcome. ELN 2022 AML guidelines clearly state that pre-allo-HSCT MRD positivity was an independent poor outcome factor after allo-HSCT. Thus, it was important to improve the CR rate and depth of remission in R/R AML patients and successfully bridge to allogeneic allo-HSCT (19).

Currently, there is a lack of standardized protocol available for the treatment of R/R AML, and there is an urgent need for effective treatment options to increase remission rates and improve prognosis (20–22). Venetoclax is rapidly occupying an important position in the treatment of hematologic malignancies due to its unique antitumor mechanism and excellent antitumor effect (23, 24). The VEN-HMA regimen has been recommended as the first-line treatment option for the elderly or unfit patients with newly diagnosed AML patients. Nevertheless, its efficacy remains limited in the R/R AML patient population (6, 8, 25, 26). Studies have shown that the CR/CRi rate of venetoclax monotherapy in R/R AML is approximately 20%, while in combination with HMAs, the CR/CRi rate is 11.6-46%, and the ORR is 40-70% (3, 6, 26–29). To further improve remission and long-term survival in this challenging patient population, several domestic and international teams have explored the venetoclax combination regimen. The DiNardo team published a study of VEN+FLAG-IDA in R/R AML and showed that this regimen resulted in an ORR of 72% and an MRD-negative rate of 69% in R/R AML, with a significant survival benefit and an incidence of grade ≥3 AEs of approximately 10% (1). The Daver team reported in “BLOOD” a clinical study of venetoclax-idasanutlin in R/R AML patients, showing that 55 R/R AML patients treated with the regimen had a CRc rate of 26.0%, a morphologic leukemia-free status (MLFS) rate of 12%, an MRD-negative rate (<0.1%) of 42.9%, and a median OS of 5.1 months, and AEs were dominated by controlled myelosuppression and gastrointestinal reactions (30). Further, another study of VEN-HMAs-HHT in R/R AML published by the Liu team demonstrated that the combination regimen had ORR rates of 78.1% in 96 patients, median OS of 22.1 months, and 1-year OS of 61.5%, with the highest incidence of grade ≥3 AEs being febrile granulocyte deficiency (37.4%) (31).

Previous studies at our center have shown that the DCAG regimen consisting of HMAs combined with a modified CAG regimen is effective in treating AML patients (32–34). A further multicenter clinical trial (NCT 02886559) of the chidamide-DCAG regimen in R/R AML illustrated that the regimen achieved a CR/CRi rate of 43.1% and ORR of 55.2% in R/R AML, with AEs mainly manifesting as myelosuppression. Both the DCAG regimen and venetoclax have been confirmed to be effective against tumor cells, and preclinical studies have demonstrated synergistic antitumor cell effects of venetoclax with decitabine and aclarubicin, providing a basis for further clinical trials. However, relevant studies on VEN-DCAG in R/R AML are limited (10). Therefore, our center will conduct further clinical trials of VEN-DCAG in R/R AML based on previous research.

In our study, 17 of 20 (85%) R/R AML patients treated with VEN-DCAG achieved CR/CRi, with an ORR of 90% and an MRD-negative CR rate of 76.5%. In the investigation with a median follow-up of 10.4 months, 14 patients have survived to date, and 12 of them are in disease-free survival status. Particularly, it is noteworthy that 5 of the 6 patients who died did not receive allo-HSCT, 1 patient who died after transplantation was MRD-positive before transplantation, and 1 of the 2 patients who survived relapse was MRD-positive before transplantation. Additionally, all 6 patients who died were from the refractory group, 3 of whom achieved CR after receiving VEN-DCAG and later died of disease relapse, while the other 3 did not achieve CR after receiving VEN-DCAG and died of disease progression. It should be mentioned that 2 of the 3 patients who did not achieve CR had previously received VEN-AZA and did not achieve CR, which may indicate that patients who did not achieve CR with the VEN-AZA regimen had limited benefit from the VEN-DCAG regimen. This conclusion was similarly supported by the results of the subgroup analyses regarding whether prior receipt of venetoclax affected efficacy. Our study also showed that patients with relapsed AML may benefit more from the VEN-DCAG regimen; that the MRD negative remission rate may be improved by increasing the number of regimen cycles; and that bridging allo-HSCT after achieving CR is a favorable factor for patients’ prolonged survival.

According to our study, the incidence of AEs was generally consistent with previous reports, but there was a significant improvement in the rate of complete disease remission, MRD negative rate, and ORR (30, 35–40). However, the conclusions of the subgroup analysis may be biased or over-interpreted due to the small sample size. In addition, in order to enable patients to achieve complete remission as early as possible, some patients were adjusted to the VEN-DCAG regimen when they received 1 course of failed induction at the clinical stage. Therefore, this study defined prior ≥1-course induction failure as refractory patients, which may have been a factor in the ultimately high remission rate. The study is an optimization and exploration of the treatment regimen for R/R AML, and initially verifies that the VEN-DCAG regimen may be an effective treatment option in R/R AML patients. Considering the creation of conditions for subsequent hematopoietic stem cell transplantation and realizing that a majority of the relapsed/refractory patient population is weak or intolerant to strong chemotherapy, this reinduction regimen need not overly strong, the duration of myelosuppression should not be prolonged, and should aim to target multiple genetic aspects effectively. Therefore, the combination of venetoclax and DCAG regimen compensated for the lack of pan-target anti-tumor effect in DCAG regimen, and compared with venetoclax combined with azacitidine regimen based on no increase in toxicity adverse effects, did not reduce the therapeutic response rate and MRD-negative remission rate, and reduced the duration of myelosuppression, with a high success rate of subsequent bridging hematopoietic stem cell transplantation. The limitations of this study primarily concern the small sample size and limited follow-up time. In future, the study will be further expanded to conduct a prospective, multicenter, and randomized controlled clinical trial to more comprehensively assess the efficacy and safety of this combination regimen.

## Data availability statement

The original contributions presented in the study are included in the article/**Supplementary Material**. Further inquiries can be directed to the corresponding author.

## Ethics statement

The studies involving humans were approved by the Ethics Committee of the First Medical Center of the PLA General Hospital (S2022-740-01). The studies were conducted in accordance with the local legislation and institutional requirements. Written informed consent for participation was not required from the participants or the participants’ legal guardians/next of kin in accordance with the national legislation and institutional requirements.

## Author contributions

LYF(LIU): Data curation, Writing – original draft. LYF(LI): Project administration, Supervision. ZR: Conceptualization, Project administration. YZY: Data curation, Formal Analysis. JY: Funding acquisition, Methodology, Project administration, Resources, Writing – review & editing.

## References

[1] DiNardo CD Lachowiez CA Takahashi K Loghavi S Xiao L Kadia T. Venetoclax combined with FLAG-IDA induction and consolidation in newly diagnosed and relapsed or refractory acute myeloid leukemia. J Clin Oncol (2021) 39(25):2768–78. doi: 10.1200/JCO.20.03736 PMC8407653 34043428

[2] Estey EH. Acute myeloid leukemia: 2019 update on risk-stratification and management. Am J Hematol (2018) 93(10):1267–91. doi: 10.1002/ajh.25214 30328165

[3] Konopleva M Pollyea DA Potluri J Chyla B Hogdal L Busman T. Efficacy and biological correlates of response in a phase II study of venetoclax monotherapy in patients with acute myelogenous leukemia. Cancer Discovery (2016) 6(10):1106–17. doi: 10.1158/2159-8290.CD-16-0313 PMC5436271 27520294

[4] Mihalyova J Jelinek T Growkova K Hrdinka M Simicek M Hajek R. Venetoclax: A new wave in hematooncology. Exp Hematol (2018) 61:10–25. doi: 10.1016/j.exphem.2018.02.002 29477371

[5] Pan R Hogdal LJ Benito JM Bucci D Han L Borthakur G. Selective BCL-2 inhibition by ABT-199 causes on-target cell death in acute myeloid leukemia. Cancer Discovery (2014) 4(3):362–75. doi: 10.1158/2159-8290.CD-13-0609 PMC3975047 24346116

[6] DiNardo CD Pratz K Pullarkat V Jonas BA Arellano M Becker PS. Venetoclax combined with decitabine or azacitidine in treatment-naive, elderly patients with acute myeloid leukemia. Blood (2019) 133(1):7–17. doi: 10.1182/blood-2018-08-868752 30361262 PMC6318429

[7] Pollyea DA Pratz K Letai A Jonas BA Wei AH Pullarkat V. Venetoclax with azacitidine or decitabine in patients with newly diagnosed acute myeloid leukemia: Long term follow-up from a phase 1b study. Am J Hematol (2021) 96(2):208–17. doi: 10.1002/ajh.26039 33119898

[8] Aldoss I Yang D Aribi A Ali H Sandhu K Al Malki MM. Efficacy of the combination of venetoclax and hypomethylating agents in relapsed/refractory acute myeloid leukemia. Haematologica (2018) 103(9):e404–7. doi: 10.3324/haematol.2018.188094 PMC6119155 29545346

[9] Huang J Hong M Zhu Y Zhao H Zhang X Wu Y. Decitabine in combination with G-CSF, low-dose cytarabine and aclarubicin is as effective as standard dose chemotherapy in the induction treatment for patients aged from 55 to 69 years old with newly diagnosed acute myeloid leukemia. Leuk Lymphoma (2018) 59(11):2570–9. doi: 10.1080/10428194.2018.1443328 29616840

[10] Weng G Huang J He X Xue T Yang L Zhang Y. Hypomethylating agents plus modified priming regimens compared with venetoclax-based regimens based on molecular characteristics for newly diagnosed patients with acute myeloid leukemia: a multi-center cohort study. Ann Hematol (2023). doi: 10.1007/s00277-023-05452-7 37723307

[11] Dartsch DC Schaefer A Boldt S Boldt S Kolch W Marquardt H. Comparison of anthracycline-induced death of human leukemia cells: programmed cell death versus necrosis. Apoptosis (2002) 7(6):537–48. doi: 10.1023/a:1020647211557 12370496

[12] Gong X Li L Wei H Liu B Zhou C Zhang G. A higher dose of dasatinib may increase the possibility of crossing the blood-brain barrier in the treatment of patients with philadelphia chromosome-positive acute lymphoblastic leukemia. Clin Ther (2021) 43(7):1265–1271.e1. doi: 10.1016/j.clinthera.2021.05.009 34120773

[13] Carceller F Hirsch SG Khabra K Petterson T Malik R Guerra-García P. High-dose etoposide and cyclophosphamide in adults and children with primary refractory and multiply relapsed acute leukaemias: The Royal Marsden experience. Leuk Res (2019) 85:106217. doi: 10.1016/j.leukres.2019.106217 31493701

[14] Bose P Vachhani P Cortes JE. Treatment of relapsed/refractory acute myeloid leukemia. Curr Treat Options Oncol (2017) 18(3):17. doi: 10.1007/s11864-017-0456-2 28286924

[15] Schlenk RF Müller-Tidow C Benner A Kieser M. Relapsed/refractory acute myeloid leukemia: any progress? Curr Opin Oncol (2017) 29(6):467–73. doi: 10.1097/CCO.0000000000000404 28857842

[16] Wattad M Weber D Döhner K Krauter J Gaidzik VI Paschka P. Impact of salvage regimens on response and overall survival in acute myeloid leukemia with induction failure. Leukemia (2017) 31(6):1306–13. doi: 10.1038/leu.2017.23 28138160

[17] Thol F Schlenk RF Heuser M Ganser A. How I treat refractory and early relapsed acute myeloid leukemia. Blood (2015) 126(3):319–27. doi: 10.1182/blood-2014-10-551911 25852056

[18] Ganzel C Sun Z Cripe LD Fernandez HF Douer D Rowe JM. Very poor long-term survival in past and more recent studies for relapsed AML patients: The ECOG-ACRIN experience. Am J Hematol (2018) 93(8):1074–81. doi: 10.1002/ajh.25162 PMC6699929 29905379

[19] Döhner H Wei AH Appelbaum FR Craddock C DiNardo CD Dombret H. Diagnosis and management of AML in adults: 2022 recommendations from an international expert panel on behalf of the ELN. Blood (2022) 140(12):1345–77. doi: 10.1182/blood.2022016867 35797463

[20] Caruso S De Angelis B Del Bufalo F Ciccone R Donsante S Volpe G. Safe and effective off-the-shelf immunotherapy based on CAR.CD123-NK cells for the treatment of acute myeloid leukaemia. J Hematol Oncol (2022) 15(1):163. doi: 10.1186/s13045-022-01376-3 36335396 PMC9636687

[21] Jin X Zhang M Sun R Lyu H Xiao X Zhang X. First-in-human phase I study of CLL-1 CAR-T cells in adults with relapsed/refractory acute myeloid leukemia. J Hematol Oncol (2022) 15(1):88. doi: 10.1186/s13045-022-01308-1 35799191 PMC9264641

[22] Yang X Wang J. Precision therapy for acute myeloid leukemia. J Hematol Oncol (2018) 11(1):3. doi: 10.1186/s13045-017-0543-7 29301553 PMC5755341

[23] Konopleva M Zhao S Hu W Jiang S Snell V Weidner D. The anti-apoptotic genes Bcl-X(L) and Bcl-2 are over-expressed and contribute to chemoresistance of non-proliferating leukaemic CD34+ cells. Br J Haematol (2002) 118(2):521–34. doi: 10.1046/j.1365-2141.2002.03637.x 12139741

[24] Buettner R Nguyen L Morales C Chen MH Wu X Chen LS. Targeting the metabolic vulnerability of acute myeloid leukemia blasts with a combination of venetoclax and 8-chloro-adenosine. J Hematol Oncol (2021) 14(1):70. doi: 10.1186/s13045-021-01076-4 33902674 PMC8074444

[25] Wei AH Montesinos P Ivanov V DiNardo CD Novak J Laribi K. Venetoclax plus LDAC for newly diagnosed AML ineligible for intensive chemotherapy: a phase 3 randomized placebo-controlled trial. Blood (2020) 135(24):2137–45. doi: 10.1182/blood.2020004856 PMC7290090 32219442

[26] Wei AH Strickland SA Jr Hou JZ Fiedler W Lin TL Walter RB. Venetoclax combined with low-dose cytarabine for previously untreated patients with acute myeloid leukemia: results from a phase ib/II study. J Clin Oncol (2019) 37(15):1277–84. doi: 10.1200/JCO.18.01600 PMC6524989 30892988

[27] DiNardo CD Rausch CR Benton C Kadia T Jain N Pemmaraju N. Clinical experience with the BCL2-inhibitor venetoclax in combination therapy for relapsed and refractory acute myeloid leukemia and related myeloid Malignancies. Am J Hematol (2018) 93(3):401–7. doi: 10.1002/ajh.25000 29218851

[28] Aldoss I Yang D Pillai R Sanchez JF Mei M Aribi A. Association of leukemia genetics with response to venetoclax and hypomethylating agents in relapsed/refractory acute myeloid leukemia. Am J Hematol (2019) 94(10):E253–5. doi: 10.1002/ajh.25567 PMC8855495 31259427

[29] Bewersdorf JP Giri S Wang R Williams RT Tallman MS Zeidan AM. Venetoclax as monotherapy and in combination with hypomethylating agents or low dose cytarabine in relapsed and treatment refractory acute myeloid leukemia: a systematic review and meta-analysis. Haematologica (2020) 105(11):2659–63. doi: 10.3324/haematol.2019.242826 PMC7604631 33131256

[30] Daver NG Dail M Garcia JS Jonas BA Yee K Kelly KR. Venetoclax and idasanutlin in relapsed/refractory AML: a nonrandomized, open-label phase 1b trial. Blood (2023) 141(11):1265–76. doi: 10.1182/blood.2022016362 PMC10651777 36265087

[31] Jin H Zhang Y Yu S Du X Xu N Shao R. Venetoclax combined with azacitidine and homoharringtonine in relapsed/refractory AML: A multicenter, phase 2 trial. J Hematol Oncol (2023) 16(1):42. doi: 10.1186/s13045-023-01437-1 37120593 PMC10149010

[32] Gao XN Su YF Li MY Jing Y Wang J Xu L. Single-center phase 2 study of PD-1 inhibitor combined with DNA hypomethylation agent + CAG regimen in patients with relapsed/refractory acute myeloid leukemia. Cancer Immunol Immunother (2023) 72(8):2769–82. doi: 10.1007/s00262-023-03454-y PMC10991359 37166484

[33] Wang L Luo J Chen G Fang M Wei X Li Y. Chidamide, decitabine, cytarabine, aclarubicin, and granulocyte colony-stimulating factor (CDCAG) in patients with relapsed/refractory acute myeloid leukemia: a single-arm, phase 1/2 study. " Clin Epigenet (2020) 12(1):132. doi: 10.1186/s13148-020-00923-4 PMC7466805 32873343

[34] Jing Y Jin X Wang L Dou L Wang Q Yao Y. Decitabine-based chemotherapy followed by haploidentical lymphocyte infusion improves the effectiveness in elderly patients with acute myeloid leukemia. Oncotarget (2017) 8(32):53654–63. doi: 10.18632/oncotarget.11183 PMC5581138 28881839

[35] Daver N Perl AE Maly J Levis M Ritchie E Litzow M. Venetoclax plus gilteritinib for FLT3-mutated relapsed/refractory acute myeloid leukemia. J Clin Oncol (2022) 40(35):4048–59. doi: 10.1200/JCO.22.00602 PMC9746764 35849791

[36] Zeidan AM Ridinger M Lin TL Becker PS Schiller GJ Patel PA. A phase ib study of onvansertib, a novel oral PLK1 inhibitor, in combination therapy for patients with relapsed or refractory acute myeloid leukemia. Clin Cancer Res (2020) 26(23):6132–40. doi: 10.1158/1078-0432.CCR-20-2586 32998961

[37] DiNardo CD Maiti A Rausch CR Pemmaraju N Naqvi K Daver NG. 10-day decitabine with venetoclax for newly diagnosed intensive chemotherapy ineligible, and relapsed or refractory acute myeloid leukaemia: a single-centre, phase 2 trial. Lancet Haematol (2020) 7(10):e724–36. doi: 10.1016/S2352-3026(20)30210-6 PMC7549397 32896301

[38] Perl AE Martinelli G Cortes JE Neubauer A Berman E Paolini S. Gilteritinib or chemotherapy for relapsed or refractory FLT3-mutated AML. N Engl J Med (2019) 381(18):1728–40. doi: 10.1056/NEJMoa1902688 31665578

[39] Daver N Garcia-Manero G Basu S Boddu PC Alfayez M Cortes JE. Efficacy, safety, and biomarkers of response to azacitidine and nivolumab in relapsed/refractory acute myeloid leukemia: A nonrandomized, open-label, phase II study. Cancer Discovery (2019) 9(3):370–83. doi: 10.1158/2159-8290.CD-18-0774 PMC6397669 30409776

[40] Ho AD Lipp T Ehninger G Illiger HJ Meyer P Freund M. Combination of mitoxantrone and etoposide in refractory acute myelogenous leukemia–an active and well-tolerated regimen. J Clin Oncol (1988) 6(2):213–7. doi: 10.1200/JCO.1988.6.2.213 3422260

