# Supplementary material for: Venetoclax combined with hypomethylating agents and the CAG regimen in relapsed/refractory AML: a single-center clinical trial
Source: Front Immunol. 2023 Nov 20;14:1269163. doi: 10.3389/fimmu.2023.1269163 (PMC10694223; doi:10.3389/fimmu.2023.1269163)
Supplement: Supplementary file 1 [file Table_1.docx]

| **Supplementary Table S1 Clinical characteristics and efficacy of 20 R/R AML patients treated with VEN-DCAG** | | | | | | | | | | | |
| --- | --- | --- | --- | --- | --- | --- | --- | --- | --- | --- | --- |
| **NO.** | **Sex** | **Age** | **Type** | **Genetic**  **feature** | **Method and efficacy** | **VEN-DCAG cycle** | **Efficacy (1st)** | **Efficacy (total)** | **Follow-up time (months)** | **Whether or not bridged to HSCT** | **End of follow-up status** |
| **Refractory group** | | | | | | | | | | | |
| 1 | Male | 10 | M2 | AML1-ETO | CDCG(PR)→DAE(NR)→VEN-DCAG(MRD+CR) | 1 | MRD+CR | - | 21.8 | Yes | Disease-free survival |
| 2 | Female | 31 | AML | MLL-AF6 | IA(NR)→VEN-AZA-CAG(MRD+CR)→VEN-AZA-CAG(MRD-CR)→VEN-AZA-CAG(MRD-CR)→VEN-AZA-CAG(MRD-CR)→VEN-AZA-CAG(MRD-CR) | 5 | MRD+CR | MRD-CR | 18.1 | No | Death |
| 3 | Male | 15 | AML | AML1-ETO、IDH2 | DAE(NR)→DCAG(PR)→VEN-DCAG(MRD+CR) | 1 | MRD+CR | - | 14.5 | Yes | Disease-free survival |
| 4 | Female | 41 | M2 | MLL-SEPTIN9、NRAS | DIA(NR)→VEN-DCAG(MRD-CR)→VEN-DCAG(MRD-CR) | 2 | MRD-CR | MRD-CR | 13.8 | Yes | Relapse survival |
| 5 | Male | 56 | M5 | IDH2、DNMT3A、WT1 | DCAG(NR)→VEN-DCAG(MRD-CR)→VEN-DCAG(MRD-CR) | 2 | MRD-CR | MRD-CR | 9.7 | Yes | Disease-free survival |
| 6 | Male | 28 | AML | IDH2、ASXL1、U2AF1 | A-AZA(NR)→VEN-DCAG(MRD-CR) | 1 | MRD-CR | - | 8.8 | Yes | Disease-free survival |
| 7 | Female | 51 | M5 | WT1、TP53 | IA(PR)→VEN-DCAG(MRD-CR)→VEN-DCAG(MRD-CR) | 2 | MRD-CR | MRD-CR | 11.2 | No | Death |
| 8 | Female | 58 | M4 | MLL-MLL | IA(NR)→VEN-DCAG(MRD-CR)→VEN-DCAG(MRD-CR) | 2 | MRD-CR | MRD-CR | 6.8 | No | Disease-free survival |
| 9 | Male | 59 | M4 | WT1、U2AF1 | DIA(NR)→VEN-DCAG(MRD+CR)→VEN-DCAG(MRD+CR) | 2 | MRD+CR | MRD+CR | 5.0 | Yes | Death |
| 10 | Female | 56 | M5 | IDH2、DNMT3A | DCAG(NR)→VEN-AZA-CAG(MRD+CR)→VEN-DCAG(MRD-CR) | 2 | MRD+CR | MRD-CR | 21.0 | No | Disease-free survival |
| 11 | Male | 37 | M4 | MLL-MLL、WT1、ASXL1、U2AF1、TET2、RUNX1 | VEN-DHAG(PR)→VEN-AZA-CAG(NR)→VEN-DCAG(NR) | 2 | NR | NR | 3.7 | No | Death |
| 12 | Male | 20 | AML | TP53、EP300 | AZA-CAG(NR)→VEN-DCAG(PR) | 1 | PR | - | 0.9 | No | Death |
| 13 | Male | 70 | AML | MLL-AF6 | VEN-AZA(NR)→VEN-AZA(NR)→VEN-DAC-HHT(NR)→VEN-DCAG(NR) | 1 | NR | - | 0.7 | No | Death |
| **Relapse group** | | | | | | | | | | | |
| 14 | Female | 50 | M2 | NMP1 | DIA(MRD-CR)→DA(MRD-CR)→DA(MRD-CR)→A-AZA(MRD-CR)→VEN-AZA(MED-CR)→A-DAC(MRD-CR)→VEN-DCAG(MRD-CR) | 1 | MRD-CR | - | 2.6 | Yes | Disease-free survival |
| 15 | Female | 39 | M4 | FUS-ERG、DNMT3A | IAG(PR)→IA(CR)→MAE(MRD+CR)→allo-HSCT(MRD-CR)→VEN-DCAG(MRD-CR)→VEN-DCAG(MRD-CR) | 2 | MRD-CR | MRD-CR | 7.1 | Yes | Disease-free survival |
| 16 | Female | 64 | M5 | IDH2、NRAS、DNMT3A、BCORL1、BCOR | IA(NR)→DIAG(MRD+CR)→DIAG(MRD-CR)→DA(CR)→DCAG(MRD+CR)→VEN-DCAG(MRD-CR) | 1 | MRD-CR | - | 8.7 | No | Disease-free survival |
| 17 | Male | 29 | M2 | AML1-ETO、C-KIT | DA(MRD+CR)→IA(MRD+CR)→Ara-C(MRD+CR)→Ara-C(MRD+CR)→HEA(MRD+CR)→Ara-C(MRD+CR)→IA(MRD+CR)→Ara-C(MRD+CR)→HAA(MRD+CR)→VEN-DCAG(MRD+CR) | 1 | MRD+CR | - | 11.0 | Yes | Relapse survival |
| 18 | Male | 25 | AA^a^→AML | IDH1、WT1、RUNX1 | allo-HSCT(CR)→VEN-DCAG(MRD-CR)→VEN-DCAG(MRD-CR) | 2 | MRD-CR | MRD-CR | 12.7 | Yes | Disease-free survival |
| 19 | Female | 65 | M5 | IDH2、DNMT3A、BCOR、TET2 | DCAG(MRD+CR)→DCAG(MRD+CR)→DCAG(MRD-CR)→DCAG(MRD-CR)→DCAG(MRD-CR)→DCAG(MRD-CR)→DHAG(MRD-CR)→DHAG(MRD-CR)→VEN-DCAG(MRD-CR)→VEN-DCAG(MRD-CR)→VEN-DCAG(MRD-CR)→VEN-DCAG(MRD-CR) | 4 | MRD-CR | MRD-CR | 13.8 | No | Disease-free survival |
| 20 | Male | 25 | M2 | WT1、CEBPA、DNMT3A | IA(MRD+CR)→IA(MRD-CR)→HAA(MRD-CR)→HAA(MRD-CR)→IA(MRD-CR)→HAA(MRD-CR)→IA(MRD-CR)→IA(MRD-CR)→VEN-DCAG(MRD+CR)→VEN-DCAG(MRD-CR) | 2 | MRD+CR | MRD-CR | 20.9 | Yes | Disease-free survival |
| CDCG: chidamide+decitabine+cytarabine+G-CSF; DAE: daunorubicin+cytarabine+etoposide; IA: idarubicin+cytarabine; DIA: decitabine+idarubicin+cytarabine; A-AZA: cytarabine+azacytidine; VEN-AZA: venetoclax+azacytidine; A-DAC: cytarabine+decitabine; IAG: idarubicin+cytarabine+G-CSF; MAE: mitoxantrone+cytarabine +etoposide; DIAG: decitabine+idarubicin+cytarabine+G-CSF; DCAG: decitabine+aclacinomycin+cytarabine+G-CSF; HEA: homoharringtonine+etoposide+cytarabine; HAA: homoharringtonine+cytarabine+aclarubicin; DHAG: decitabine+homoharringtonine+cytarabine+G-CSF; AA^a^: Aplastic anemia | | | | | | | | | | | |
